# Analysis of Treatment Influence on Kidney Function and Brain Non-Contrast MRI Vascular Manifestations of Systemic ANCA-Associated Vasculitis with Renal Involvement

**DOI:** 10.3390/jcm15010058

**Published:** 2025-12-21

**Authors:** Arkadiusz Lubas, Jacek Staszewski, Ksymena Leśniak, Grzegorz Spłocharski, Arkadiusz Zegadło, Artur Maliborski, Aleksander Dębiec, Julia Bryłowska, Tymoteusz Lubas, Stanisław Niemczyk

**Affiliations:** 1 Department of Internal Diseases, Nephrology and Dialysis, Military Institute of Medicine—National Research Institute, 04-141 Warsaw, Polandsniemczyk@wim.mil.pl (S.N.); 2 Department of Neurology, Military Institute of Medicine—National Research Institute, 04-141 Warsaw, Poland; 3 Department of Radiology, Military Institute of Medicine—National Research Institute, 04-141 Warsaw, Poland; 4 Faculty of Medicine, University of Warsaw, 02-089 Warsaw, Poland; j.brylowska@student.uw.edu.pl; 5 Faculty of Medicine, Medical University of Gdańsk, 80-210 Gdańsk, Poland

**Keywords:** cerebral vasculitis, antineutrophil cytoplasmic antibodies, white matter lesions, immunosuppression

## Abstract

**Background**: Antineutrophil cytoplasmic antibody-associated systemic vasculitis (AAV) most often involves the kidneys, upper airways and lungs, and peripheral and central nervous systems (PNS, CNS). However, in contrast to PNS, the involvement of the CNS is rarely taken into account in the recognition and assessment of systemic vasculitis, probably because of nonspecific symptoms such as headaches and dizziness, aphasia, memory disorders, or mood changes. In addition, it is not clear whether treatment of systemic vasculitides reduces cerebral vascular alterations. In this study, we aimed to evaluate the effects of AAV treatment on vascular and vasogenic alterations in the brain in patients with acute vasculitis onset with renal involvement. **Methods**: Twenty-nine patients (17F, 12M, age 60.4 ± 9.8) with AAV relapse with renal involvement were included in the study. The initial baseline assessment and the second evaluation, performed 12.6 ± 2.5 months after the beginning of immunosuppressive treatment, included clinical, neurological, and renal function assessments, along with a brain MRI. **Results**: Compared with baseline, improvement in clinical, neurological, and renal function was observed during the second clinical evaluation. A significant reduction in the occurrence of vascular dilatation and narrowing in secondary (37.9% vs. 17.2%; *p* = 0.031) and tertiary (37.9% vs. 10.3%; *p* = 0.008) cerebral vascular branches was observed. However, the number of vasogenic cerebral white matter lesions detected on the FLAIR sequence increased significantly (36.0 vs. 48.0%; *p* < 0.001). **Conclusions**: Intensive immunosuppressive treatment of acute-onset systemic AAV with renal involvement decreases disease activity, improves kidney function, and decreases central nervous system vascular but not vasogenic alterations.

## 1. Introduction

Antineutrophil cytoplasmic antibody (ANCA)-associated vasculitides (AAV) are a heterogeneous group of rare autoimmune systemic diseases characterized by necrotizing inflammation of small- and medium-sized blood vessels. Three conditions that fall under the definition of AAV are microscopic polyangiitis (MPA), granulomatosis with polyangiitis (GPA), and eosinophilic granulomatosis with polyangiitis (EGPA). MPA, driven mainly by myeloperoxidase antibodies (p-ANCA), is in most cases limited to the kidneys, whereas both GPA, in about 75% associated with proteinase-3 antibodies (c-ANCA), and EGPA, induced by eosinophiles and in 40% by ANCA, are usually expressed in the upper airways and lungs, with about 70% renal involvement in GPA and 25% in EGPA [1,2]. Although secondary nervous system involvement is not uncommon in all types of AAV, it is usually the peripheral nervous system (PNS) that is affected, with relatively low central nervous system (CNS) involvement, reported in up to 15% cases [3,4]. However, the proportions vary among the different types of AAV, as PNS is more often affected in MPA and EGPA than in GPA, whereas CNS involvement is more common in GPA, with rare occurrence in MPA and EGPA [5]. Meningitis, pituitary gland granulomatous alterations, and vasculitis are the three ways in which AAV can affect the CNS; the last is the most common, and the others are rare in GPA and even rarer in MPA or EGPA. Diagnosis of AAV is in many cases challenging due to its various and nonspecific clinical symptoms, often overlapping with other autoimmune disorders [6]. Microscopic hematuria and proteinuria, rapid kidney function decline, and the presence of ANCA allow the diagnosis of AAV with renal involvement (AAVR). However, the detection of ANCA in an antigen-specific assay alone cannot be considered diagnostic, and a negative result does not exclude AAV [7]. Diagnosis of CNS involvement in AAV is based on clinical assessment, followed by radiologic imaging. The clinical manifestations are not specific and include headaches, aphasia, seizures, deterioration of cognitive functions, altered consciousness, coma, and others, the onset of which may be acute, subacute, or chronic. Nonspecific and less severe symptoms of brain vasculitis may be overlooked in the diagnostic process and misclassified as symptoms of a systemic disease, especially since cerebral vasculitis is not a standard criterion for assessing AAV activity. Nevertheless, analyses of prospective studies indicate a high incidence of cerebral vasculitis—up to 40%—in ANCA-associated systemic vasculitis [2]. Despite this, data on the effectiveness of immunosuppressive therapy of inflammatory changes in cerebral vessels in the course of systemic ANCA-associated vasculitis are limited. In radiologic tests, especially magnetic resonance imaging sequences such as susceptibility-weighted angiography (SWAN) or fluid-attenuated inversion recovery (FLAIR), alterations in the lumen of cerebral vessels—such as longitudinal narrowing or alternating narrowing and dilatation—resulting from the inflammatory process can be detected, as can white matter lesions (VWML). In general, to date, the presence of secondary brain vasculitis does not influence treatment recommendations for AAV as a systemic disease. Induction therapy with corticosteroids and immunosuppressants is needed to achieve remission and prevent relapses [8]. Immunosuppressants most commonly used in such treatment are cyclophosphamide or rituximab, which have been demonstrated to have superior remission rates for the c-ANCA subgroup of AAV.

The study aimed to evaluate the effectiveness of immunosuppressive treatment inducing remission of ANCA-associated systemic vasculitis with renal involvement on the occurrence of brain vascular and vasogenic lesions.

## 2. Materials and Methods

We recruited participants from August 2019 to November 2021, with informed consent obtained, as described earlier [2]. However, the presented study included patients with severe AAVR flare who had already undergone immunosuppressive induction therapy followed by maintenance treatment. Age ≥ 18 years, double assessment of brain changes in MRI before and after intensive treatment, and a signed informed consent were prerequisites for the study. Exclusion criteria included disturbances of consciousness or poor clinical condition preventing the signing of informed consent or proper performance of MRI, contraindications to brain MRI (e.g., metal implants, claustrophobia), active oncological condition or symptoms of active viral or bacterial infection disqualifying from immunosuppressive treatment, and advanced impairment corresponding to the need to remain in bed for more than half of the waking time. Moreover, patients who had only one brain MRI assessment were excluded.

### 2.1. Clinical Evaluation

The Birmingham Vasculitis Activity Score for Wegener’s granulomatosis (BVAS/WG) was used to assess AAV activity based on available medical records, interviews, physical examination, and test results [9]. In this study, we included only patients who experienced a severe disease flare, as defined by the BVAS/WG evaluation form: the occurrence of ≥1 new/worsening major symptom. The items concerning nervous system involvement are included in the BVAS/WG scale as major items, so they may or may not have been present at the time of qualification for treatment of patients included in the study. Nevertheless, in the presented study, the minimal BVAS/WG score at inclusion was 3.

### 2.2. Laboratory Tests

Blood tests were used to measure hemoglobin (Hgb), as well as the concentrations of creatinine, urea, antineutrophil antibodies p-ANCA and c-ANCA [IU/mL], and highly sensitive C-reactive protein (hsCRP). Furthermore, the second morning urine spot sample was used to quantify the urinary albumin-creatinine ratio (UACR) [mg/g]. Hemoglobin concentrations were measured using automated hematological analysis with the XN-1000 analyzer (SYSMEX Corporation, Kobe, Japan, 2018). Creatinine concentrations (serum and urine) and serum urea were determined by enzymatic and kinetic urease methods, respectively, on the Cobas c 501 analyzer (Roche Diagnostics, Rotkreuz, Switzerland, 2019). Urine albumin and hsCRP were quantified by nephelometry using the nephelometer BN II (SIEMENS Healthcare Diagnostics Products GmbH, Marburg, Germany, 2017). p-ANCA and c-ANCA were assessed via fluorimetric enzyme-linked immunoassay (FEIA) on the Unicap 100 analyzer (HVD HOLDING AG, Warsaw, Poland, 2000). UACR was calculated using the parameters detailed in the following section.

### 2.3. Neurological Evaluation

Based on neurological examinations, diagnoses, and reported symptoms, a structured medical history was obtained. History of stroke and transient ischemic attack (TIA), mono- or polyneuropathy, myopathy, persistent headaches, abnormal gait, seizures, sensory loss, encephalopathy, and muscular weakness were all given special consideration. Chronic headache was defined according to ICHD-3 criteria as headache occurring on ≥15 days per month for at least 3 consecutive months, irrespective of etiology [10]. The same two board-certified senior neurologists performed a neurological physical examination at baseline and at stage 1, which included an assessment of both the central nervous system and the peripheral nervous system. Assessments of the cranial nerves, reflexes, motor system, coordination, and gait were all part of it. What is more, peripheral nervous system dysfunction was diagnosed in cases of peripheral mononeuropathy, multiple mononeuropathy, polyneuropathy, or cranial neuropathy symptoms, whereas central nervous system dysfunction was diagnosed in cases of corticospinal, brainstem, or cerebellar symptoms.

### 2.4. Magnetic Resonance Imaging of the Brain

A 3.0 Tesla scanner (GE Discovery MR 750W Waukesha, WI, USA, with a 12-channel head coil) was used to perform Brain MRI at Stage 0 and Stage 1 after completion of AAV induction treatment. It was performed by using several commonly available techniques: black blood (BB) (pulse-gated (PG), short tau inversion recovery (STIR), fast spin echo (FSE), axial), time-of-flight (TOF), diffusion-weighted imaging (DWI), axial T2 FLAIR FSE, SWAN, sagittal 3D Cube T2 Iso FSE, and sagittal 3D Cube T1 Iso FSE, without the use of contrast. All of these methods have been recommended for the assessment of the brain and vessels [11]. Imaging parameters for the sequences used have been described previously [2]. Two senior certified radiologists independently reviewed the obtained images. The vessel lumen may be identified using the BB technique, which shows blood in the vessels in a black color. Assessing the vessel lumen contour can be achieved thanks to the 3D TOF angiography. This examination, performed in a 1.5 Tesla MRA, is comparable to digital subtraction angiography [12]. MRI vasculitis indicators such as focal and longitudinal stenosis, alternating vasoconstriction and dilatation (VAND), and stenoses were found and quantified. Focal, hyperintense T2-weighted white matter lesions (VWML) and low signal intensity hemosiderin deposits on SWAN images were recognized as vascular lesions.

### 2.5. Treatment

In the present study, patients were not eligible for treatment, but those who had already undergone immunosuppressive induction therapy were included. All participants in the study received immunosuppressive induction treatment followed by maintenance therapy in accordance with the applicable KDIGO guidelines [13]. Remission induction therapy, in the form of intravenous cyclophosphamide (CYC) associated with glucocorticosteroids (GS) in tapered doses, was administered to twenty-three patients. In two patients, CYC doses were reduced to 6 for clinical reasons, and four patients received intravenous rituximab. Additionally, before starting induction treatment, six patients underwent 3 to 10 therapeutic plasma exchange procedures. In maintenance therapy, after induction, azathioprine with GS (20 patients), mycophenolate mofetil with GS (4 patients), rituximab (3 patients), or GS alone (2 patients) were used.

### 2.6. Statistical Analysis

Results were presented as mean with standard deviation (SD) or median with interquartile range (IQR), depending on whether the data followed a normal distribution, which was checked with the Shapiro–Wilk test. Nominal data were presented as numbers with percentage occurrence. Differences between normally distributed variables were investigated using a *t*-test for dependent data, otherwise with the Wilcoxon test. Nominal variables were compared with a chi-squared test or an exact F test if the number of observations was <6 in any one group. The significance of the test was considered if the two-tailed *p*-value was below 0.05. Based on the analysis of the results of the number of VWML performed in the first 6 patients (median 12.0, IQR 17.0 vs. median 13, IQR 22; *p* = 0.043) in whom brain MRI was assessed in 2 stages, the analysis of the target group size showed that to obtain the power of the test of 80%, the size of the study group should be equal 26. For statistical analysis, the Tibco Statistica v. 13.3 (TIBCO Software Inc., Greenwood Village, CO, USA) package was used.

## 3. Results

Of the initially recruited thirty-eight patients with acute-onset ANCA-associated vasculitis with renal involvement, 5 died, 2 did not consent to another brain MRI examination, and 2 did not report for continuation of treatment. Finally, twenty-nine patients (17F, 12M, age 60.4 ± 9.8; 23 with predominant p-ANCA, 6 with c-ANCA), treated intensively with immunosuppressants and continuing these agents as maintenance therapy, were included in this study. The second evaluation was performed at Stage 1, 12.6 ± 2.5 months after the initial assessment. A comparison of disease activity and blood and urine test results between the two evaluations is presented in Table 1.

After the ordered treatment, a significant decrease in clinical disease activity, inflammatory markers, and specific ANCA concentration was observed. Moreover, a substantial improvement in kidney function was indicated. From the entire group, six [20.7%] patients were treated with hemodialysis (HD) at the time of inclusion, from which only three [10.3%] continued this therapy in Stage 1. However, two patients without HD in Stage 0 required hemodialysis in Stage 1. The change in hemodialyzed patients between stages was not significant (20.7 vs. 17.2%; *p* = 0.655). The results of the neurological assessments are shown in Table 2.

Neurological investigations revealed that most neurological alterations were stable and did not change during observation. However, substantial improvement in polyneuropathy and headaches was noted.

### 3.1. Vascular Alterations

**Figure 1:**
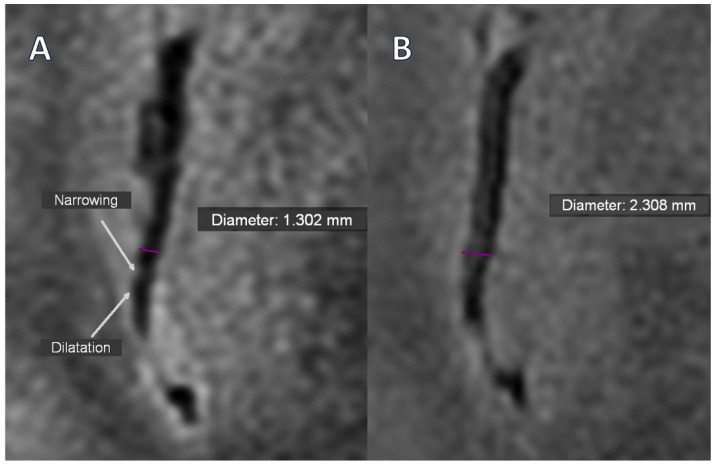
The intracranial vessel in BB STIR T2 axial sequence; Purple—diameter; (**A**)—Alternating narrowing and dilatation (white arrows) visible before treatment; (**B**)—Normalization of vessel lumen after treatment (Stage 1).

**Figure 2:**
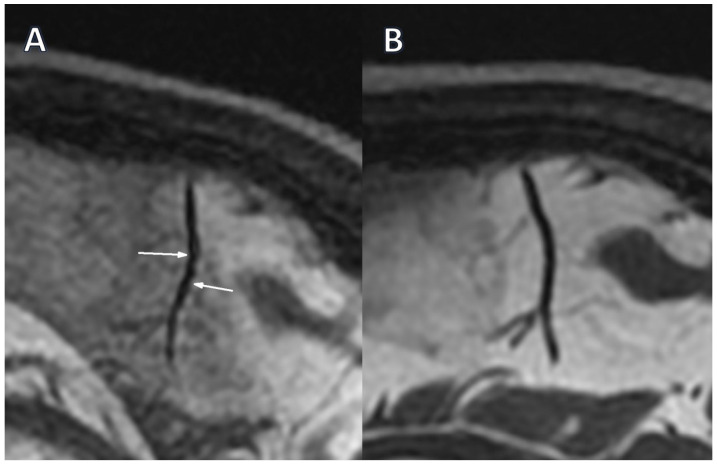
The intracranial vessel in the sagittal plane in the BB STIR T2 sequence. (**A**)—Alternating narrowing and dilatation (white arrows) before treatment (Stage 0); (**B**)—Normalization of vessel lumen after treatment (Stage 1).

Only two segmental arterial narrowing in secondary vascular branches were investigated in Stage 0 TOF, which were absent in Stage 1 (Table 3). However, this change was not substantial. The occurrence of VAND in secondary as well as in tertiary vascular branches was initially detected in the BB sequence in 37.9% of patients, with their reduction to 17.2% (*p* = 0.031) and 10.3% (*p* = 0.008) in Stage 1, respectively (Figure 1A,B, Figure 2A,B).

There were no significant differences in arterial narrowing or VAND between participants treated with cyclophosphamide or rituximab in either stage.

### 3.2. Vasogenic Lesions

The vasogenic lesions in the cerebral white matter (VWML) were detected in the majority of patients in Stage 0 and did not change substantially in Stage 1 (Table 4). However, the number of VWML detected by the FLAIR technique increased significantly in Stage 1 compared with the initial assessment.

There were no significant differences in the number of VWML between participants treated with cyclophosphamide or rituximab in both stages.

## 4. Discussion

Our investigation shows that therapy with glucocorticosteroids and immunosuppressants, such as cyclophosphamide or rituximab, has a significant impact on kidney function and brain vascular manifestations of systemic ANCA-associated vasculitis in patients with acute disease onset. In the available scientific literature, we did not find any similar studies that prospectively assessed the effect of immunosuppressive therapy on vascular and vasculogenic changes in the brain in patients with relapse of systemic ANCA-dependent vasculitis with renal involvement. However, our findings are in line with current treatment strategies in AAV, where intensive induction therapy with corticosteroids and immunosuppressants such as cyclophosphamide or rituximab leads to remission within 8–12 weeks, after which patients are switched to maintenance regimens with azathioprine, methotrexate, mycophenolate mofetil, or rituximab to prevent relapses [9].

The presented results show that renal parameters improved, disease activity decreased, and concomitant ANCA titers decreased, with CNS vascular alterations reduced after intensive immunosuppression. We noticed that, in the Black-Blood sequence, the incidence of alternating stenosis and dilation (VAND), a marker of vasculitis, in secondary and tertiary vessels decreased from 37.9% to 17.2% (*p* = 0.031) and to 10.3% (*p* = 0.008), respectively. This indicates that cerebral vascular changes in the course of AAV may significantly improve after intensive immunosuppressive treatment. In this regimen, in addition to high but tapered doses of glucocorticosteroids, intravenous infusions of cyclophosphamide or rituximab were administered, and we did not find significant differences in arterial narrowing or VANDs between participants treated with cyclophosphamide or rituximab in either stage. These observations support the interpretation that VANDs are sensitive to resolution in time of intensive active vasculitis treatment. Moreover, focusing on active cerebral vessels alterations, our results are consistent with the RAVE study, which demonstrated that rituximab was non-inferior to cyclophosphamide in inducing remission in severe forms of AAV, and the RITUXVAS study, which confirmed comparable efficacy of both drugs in patients with renal involvement [14,15]. Our data also align with the current EULAR guidelines, which recommend the use of high-dose glucocorticosteroids in combination with rituximab or cyclophosphamide as first-line treatment to achieve remission [16].

Similar observations were also reported in studies based on vessel wall MRI. Wagner et al. demonstrated that in patients with primary vasculitis of the central nervous system, vessel wall enhancement (VWE) systematically decreased during the first year after initiation of immunosuppression and, in some cases, completely resolved [17]. The convergence of these results with our observation of reduced VANDs in Black-Blood and TOF sequences suggests that brain vasculitis markers are sensitive to resolution during immunosuppressive treatment. It should be emphasized, however, that the presented results regarding primary vasculitis of the central nervous system refer to a distinct disease entity from ANCA-associated vasculitis, which limits direct comparison. Also, the imaging approaches were diverse because we assessed luminal changes (VAND) using non-contrast Black-Blood and TOF sequences, while Wagner focused on wall pathology (VWE) with contrast-enhanced vessel wall MRI. Nevertheless, the similarity in the dynamics of vascular changes suggests a common mechanism of inflammatory response to treatment. Performing serial high-resolution MRI vessel wall imaging assessments, Shimoyama et al. demonstrated a significant reduction in strong and concentric vessel wall enhancements after a mean 15.6-month treatment period (25.5% vs. 12.4%, *p* < 0.001) in patients with central nervous system vasculitis [18]. However, in patients with relapse, the total vessel wall enhancement score worsened, which confirms the possibility of monitoring the effectiveness of treatment of cerebral vasculitis with the imaging method. In our study, we did not observe disease recurrence in any patient, but the next imaging of cerebral vascular changes was performed one year (mean 12.6 months) after the initiation of intensive immunosuppressive treatment.

In the present study, the SWAN technique showed a decrease in VWML (*p* = 0.053), indicating a trend towards significance and suggesting that treatment was successful. Unexpectedly, in the FLAIR technique, we observed an increase in the number of VWML after 1 year of immunosuppressive treatment (21.0 (36.0) vs. 27.0 (48.0); *p* < 0.001). The differences in results between the SWAN and FLAIR sequences in this study do not indicate distinct pathophysiological mechanisms but rather reflect the different sensitivities of these techniques to specific phases of cerebral microcirculatory injury in the course of AAV. The reduction in the number of lesions observed in the SWAN sequence after treatment may reflect the resolution of active vascular inflammation and microhemorrhages, confirming the effectiveness of intensive immunosuppressive therapy. In contrast, the increase in the number of VWML detected in the FLAIR sequence, despite clinical improvement, suggests the progression of persistent consequences of prior microcirculatory damage, such as demyelination that evolves more slowly and is not promptly reversed by immunosuppression. In addition, the FLAIR sequence is a dedicated method for detecting VWML, but the SWAN sequence can help in the differential diagnosis of vascular or other VWML origins. This indicates that treatment effectively suppresses the inflammatory process but does not always prevent the development of secondary structural changes in the white matter. An increase in the number of VWML suggests increased microvascular damage. It can also indicate the deterioration of neurological functions, such as cognitive impairment, mood disorders, or reduced physical function. These clinical observations support the correlation between increased white matter lesions and increased mortality and disability [19].

The etiology of cerebral VWML is heterogeneous and includes chronic dysfunction of endothelial tight junctions, perivascular astrocytic support, and glymphatic clearance, driven by microvascular ischemic disease/small-vessel disease, atherosclerosis, migraine, vasculitis (including ANCA-associated), and drug-related leukoencephalopathy (reported in ~1–4% with certain immunosuppressants) [19]. Single case reports suggest that immunosuppressive therapy is only partially effective, if at all, in VWML vasculitis. In a 33-year-old woman, who was admitted in April 2020 to the Montreal Neurological Hospital following a 4-month history of cognitive decline and personality changes, struggling with GPA, and receiving sufficient immunosuppression with rituximab, intravenous immunoglobulin, and corticosteroids, MRI showed substantial progression of diffuse T2/FLAIR hyperintense white matter lesions [20]. This underscores the validity of the study, which indicates the possibility of progression of vasogenic changes in white matter despite appropriate immunosuppression. This demonstrates that not all lesions resembling vasculitis can improve with standard treatment. However, our work provides systematic data from a broader cohort of AAV patients, whereas this case remains a single-patient observation with a potentially distinct underlying illness. On the other hand, VWML identified on MRI in our study may have a different etiology, such as age, hypertension, atherosclerosis, and not ANCA-associated systemic vasculitis. Investigating 208 patients with asymptomatic cerebral artery stenosis who were randomly assigned to simvastatin 20 mg daily, Mok et al. found no substantial change in VWML volume between the treated and placebo groups after 2 years of follow-up [21]. However, stratified analysis revealed that the increase in active VWML volume was significantly lower in the simvastatin group (*p* = 0.047). These data support the suggestion that observed VWML can have different etiologies, may reflect irreversible changes, and may progress despite, or even with, immunosuppressive treatments. On the other hand, Camard et al., studying 11 patients with ANCA-related vasculitis, found cognitive impairment in 4 (36%) patients, mainly affecting attentional and executive functions, suggesting vascular involvement, despite the absence of VAND and VWML in brain MRI [22]. In contrast to our work, in this study, most of the examined patients did not have active AAV; only 2 had BVAS scores of 1 and 5, respectively. Additionally, no Black-Blood sequence was used, which may justify the lack of VAND identification in the MRI examination.

Our observation that polyneuropathy and chronic headaches declined after induction therapy aligns with recent evidence that effective immunosuppression (high-dose glucocorticoids with rituximab or cyclophosphamide) leads to neurological improvement in vasculitic neuropathy and symptom relief as systemic inflammation remits. Contemporary guidelines and reviews report recovery of vasculitic neuropathy under such regimens, and case-level data in AAV document complete headache resolution following treatment [23,24].

Although promising results, our work has some limitations. The first one is a relatively small number of the investigated group. However, to avoid missing data, we included only patients who were evaluated after 1 year of immunosuppressive treatment and excluded those evaluated only at baseline or during treatment [2]. Secondly, a small sample size could explain the lack of differences in treatment effects across drug regimens. On the other hand, induction treatment is a complex regimen, making it difficult to draw conclusions about the effects of individual drugs on the detected vascular and vasculogenic changes in the brain. Moreover, some *p*-values reported as non-significant or at the significance level may be substantial in larger groups. Also, the long-term trajectories of cerebral vascular alterations are unknown, as follow-up was restricted to a single imaging time point after induction treatment. Moreover, due to the inclusion of patients with severe vasculitis flare with kidney involvement and unknown kidney function outcomes, we avoided gadolinium contrast media, and direct vascular wall enhancement could not be assessed. In addition, whereas Black-Blood, TOF, SWAN, and FLAIR sequences offer useful complementary data, direct comparisons between modalities are made more difficult by their varying sensitivities and specificities. To overcome the limitations outlined above and confirm the presented results, prospective studies with larger sample sizes, different treatment regimens, and longitudinal follow-up should be conducted.

## 5. Conclusions

Vascular and vasogenic alterations in the central nervous system associated with systemic ANCA-associated vasculitis can be detected and monitored with non-contrast MRI using specific sequences. Intensive immunosuppressive treatment for acute-onset systemic AAV with renal involvement improves kidney function, decreases disease activity, and reduces central nervous system vascular alterations. However, the impact of ANCA-associated vasculitis induction therapy on vasogenic white matter lesions remains unknown, and future research is needed to develop etiology-specific treatments for these brain alterations.

## Figures and Tables

**Table 1:** Results of disease activity, blood, and urine tests in both stages.

	Stage 0	Stage 1	Significance—*p*
	Mean ± SD Median (IQR) N [%]	Mean ± SD Median (IQR) N [%]	
BVAS [point]	7.6 ± 3.1	1.0 (1.0)	<0.001
Hgb [g/dL]	10.2 ± 1.8	11.8 ± 1.8	<0.001
Creatinine [mg/dL]	2.9 (1.9)	1.8 (1.4)	0.049
Urea [mg/dL]	102.4 ± 47.4	57.0 (48.0)	<0.001
hsCRP [mg/dL]	0.85 (1.30)	0.30 (0.50)	0.010
UACR [mg/g]	1077.4 (2349.6)	421.7 (863.5)	0.161
p-ANCA [IU/mL]	81.5 (114.0)	3.9 (17.8)	<0.001
c-ANCA [IU/mL]	80.5 (55.0)	28.0 (41.0)	0.043

BVAS—Birmingham Vasculitis Activity Score for Wegener’s granulomatosis, c-ANCA—neutrophil proteinase 3 antineutrophilic cytoplasmic antibody; p-ANCA—neutrophil myeloperoxidase antineutrophilic cytoplasmic antibody; hsCRP—high-sensitive C-reactive protein; UACR—urinary albumin/creatinine ratio.

**Table 2:** Results of the neurological assessment.

	Stage 0	Stage 1	Significance—*p*
	Number (%)	Number (%)	
Chronic headaches *	7 [24.1]	4 [13.8]	0.083
Gait imbalance *	8 [27.6]	7 [24.1]	0.564
Epilepsy *	0 [0.0]	0 [0.0]	-
Legs and/or arms paresthesia *	8 [27.6]	9 [31.0]	0.564
Cerebral stroke or TIA *	0 [0.0]	0 [0.0]	-
Cranial neuropathy	1 [3.4]	2 [6.9]	0.317
Mononeuritis/Mononeuritis multiplex	10 [34.5]	6 [20.7]	0.102
Polyneuropathy	11 [37.9]	4 [13.8]	0.020
Ataxia	13 [44.8]	11 [37.9]	0.414
Pyramidal symptoms	6 [20.7]	6 [20.7]	1.000
Extra-pyramidal symptoms **	7 [24.1]	7 [24.1]	1.000
CNS dysfunction	7 [24.1]	5 [17.2]	0.317
PNS dysfunction	13 [44.8]	14 [48.3]	0.655

CNS—central nervous system; PNS—peripheral nervous system; TIA—transient ischemic attack; * in anamnesis, ** hand tremor.

**Table 3:** Comparison of the cerebral MRI angiography results in both stages.

	Sequence	Stage 0	Stage 1	*p*-Value
		Number [%]	Number [%]	
Segmental narrowing in secondary vascular branches	BB	1 [3.4]	1 [3.4]	1.000
	TOF	2 [6.9]	0 [0.0]	0.157
Segmental narrowing in tertiary vascular branches	BB	0 [0.0]	0 [0.0]	-
	TOF	0 [0.0]	0 [0.0]	-
Alternating narrowing and dilatation in secondary vascular branches	BB	11 [37.9]	5 [17.2]	0.014
	TOF	1 [3.4]	0 [0.0]	0.317
Alternating narrowing and dilatation in tertiary vascular branches	BB	11 [37.9]	3 [10.3]	0.005
	TOF	1 [3.4]	0 [0.0]	0.317

BB—Black Blood, TOF—Time of Flight.

**Table 4:** Comparison of the occurrence and quantity of vasogenic white matter lesions in FLAIR and SWAN MRI techniques in both stages.

	Sequence	Stage 0	Stage 1	*p*-Value
		Median (IQR) Number [%]	Median (IQR) Number [%]	
Presence of white matter vasogenic lesions	FLAIR	27 [93.1]	28 [96.6]	0.564
	SWAN	27 [93.1]	26 [89.7]	0.564
Number of white matter vasogenic lesions	FLAIR	21.0 (36.0)	27.0 (48.0)	<0.001
	SWAN	25.0 (39.0)	23.5 (48.0)	0.053

FLAIR—Fluid-Attenuated Inversion Recovery sequence, SWAN—Susceptibility-Weighted Angiography.

## Data Availability

The data presented in this study are available on request from the corresponding author.
